# Negative Impact on Growth and Photosynthesis in the Green Alga *Chlamydomonas reinhardtii* in the Presence of the Estrogen 17α-Ethynylestradiol

**DOI:** 10.1371/journal.pone.0109289

**Published:** 2014-10-13

**Authors:** Tessa Pocock, Stefan Falk

**Affiliations:** Department of Natural Sciences, Mid Sweden University, Sundsvall, Sweden; Royal Netherlands Institute of Sea Research (NIOZ), Netherlands

## Abstract

It is well known that estrogenic compounds affect development of fertilized eggs of many species of birds, fish and amphibians through disrupted activity of carbonic anhydrase (CA). The most potent activity comes from the most commonly occurring synthetic sterol, 17α-Ethynylestradiol (EE2). Less is known about the responses of aquatic phytoplankton to these compounds. Here we show for the first time that, in comparision to the control, the addition of 7 µM EE2 reduced the growth rate of the green alga *Chlamydomonas reinhardtii* by 68% for cells grown at high CO_2_. When cells were grown in ambient air (low C_i_) with a fully activated carbon concentrating mechanism through the induction of CA activity, the growth rates were reduced by as much as 119%. A reduced growth rate could be observed at EE2 concentrations as low as 10 pM. This was accompanied by a reduced maximum capacity for electron transport in photosystem II as determined by a lower F_V_/F_M_ for low C_i_-grown cells, which indicates the involvement of CAH3, a CA specifically located in the thylakoid lumen involved in proton pumping across the thylakoid membranes. These results were in agreement with an observed reduction in the chloroplastic affinity for C_i_ as shown by a strong increase in the Michaelis-Menten K_0.5_ for HCO_3_^−^. In itself, a lowering of the growth rate of a green alga by addition of the sterol EE2 warrants further investigation into the potential environmental impact by the release of treated waste water.

## Introduction

Many chemical compounds released into our environment can be classified as endocrine disrupters (EDs), substances that have the capacity to adversely alter animal endocrine functions [1] leading to changes in growth, reproduction, development or behavior [2]. Often the substances are diffusely entering the environment but in other cases the source is well known as, for example, from waste water. The primary synthetic estrogen in typical oral contraceptives used by more than 100 million women worldwide [3] is the sterol 17α-Ethynylestradiol (EE2). Up to 80% of the EE2 consumed is excreted as un-metabolized conjugates in urine. The bioaccumulation of EE2 in waste water plants has been shown to impact aquatic life with the main focus being on fish [4]. The contribution of EE2 to the total amount of excreted estrogens is only about 1% but this compound is considerably more persistent in sewage effluents as compared to the naturally occurring hormones such as 17β-Estradiol (E2) and estrone (E1) [5]. In *in vivo* studies in fish, EE2 was shown to be 11-130 times more potent than E2, which in turn was 2.3–3.2 times more potent than E1 ([4], [6]–[8], see [5] for a review). EE2 is also considered to be more hydrophobic [4]. Removal of estrogens from sludge in sewage treatment plants is dependent on temperature, where warm summer temperatures would successfully eliminate EE2 [4]. However, many areas at northern European latitudes have comparatively short summer seasons and in remote areas in e.g. Sweden, the typical sewage treatment is performed by open oxidation ponds which results in lower EE2 removal/degradation during a large part of the year [4], [9].

It has been shown that estrogens can affect egg hatching success and embryonic growth of frogs and eggshell formation in birds. Eggshell production in birds was affected as a result of disrupted carbonic anhydrase (CA) activity [10]–[13]. Carbonic anhydrases are present in several locations in green algae; in the periplasmic space, the plasma membrane, mitochondria, the chloroplast envelope and stroma as well as in the thylakoid membranes [14]. CAs play an important role in the bidirectional conversion of CO_2_ to HCO_3_^−^ as part of the inorganic carbon concentration mechanism (CCM) but lately more emphasis has been focused on the involvment of CAs in the activity of the water splitting enzyme and the transfer of electrons to photosystem II [14], [15]. *Chlamydomonas reinhardtii* is a green alga that is widely used as a model system for studies in plant cell physiology. This species has an unexpectedly large number of CAs, up to ten putative CA genes are found encoded in its genome, representing all three major CA evolutionary lineages [15]. The α-type thylakoid lumen located carbonic anhydrase (CAH3) plays an essential role in the rapid dehydration of the accumulated HCO_3_^−^ to release CO_2_ into the pyrenoid, the Rubisco-containing internal compartment of the chloroplast where CO_2_ is assimilated [16]–[18]. CAH3 was also found to be functionally associated to the donor side of photosystem (PS) II, the site of proton release and the production of O_2_ from water [14], and is the only CA shown to be an essential component of the CCM [15]. Here we describe the effects of the synthetic estrogen EE2 on CA activity and its localization in *C. reinhardtii* through the use of specific inhibitors of the CAs.

## Material and Methods

### Culture conditions

Cells of *Chlamydomonas reinhardtii*, strain UTEX 89, were grown in 200 mL batch cultures as described in [19] or 400 mL turbidostat tubes at a chlorophyll concentration of 5 µg mL^−1^. Nutrients were provided by Bold's Basal Medium (BBM) and the cultures were kept at a temperature of 25°C and an irradiance of 150 µmol quanta m^−2^ s^−1^ photosynthetically active radiation (PAR) provided by fluorescent warm-white light banks (Philips TLD 18W/830). The low level of CO_2_-supply (low-C_i_) to the culture was provided by slow bubbling of air at a rate of 25 mL min^−1^ while the high CO_2_-condition (high-C_i_) was created by bubbling 2.5% CO_2_ in air as above. Batch cultures were grown only in low C_i_ conditions and were exposed to 17α-ethynylestradiol (EE2) at concentrations ranging from 10 pM to 10 µM by adding this at the same time as the tubes were inoculated with the algae. The turbidostat-grown cultures received the EE2 (7 µM) by adding this to the tank of the complete BBM-solution and the cultures were allowed to grow through at least 2 volumes of the turbidostat tube to result in a complete switch to fully estrogen-exposed cultures.

### Analytical methods

The relative growth rates were determined as the doubling time by measurement of change in A_750_ as in [19] for the batch cultures (inoculation optical density 0.028±0.001) or by collecting the overflow from the turbidostat culture over at least 24 h. Since the turbidostat is designed to keep a constant concentration of cells in the culture tube and that growth per definition is maintained continuously at the maximum growth rate, μ_max_, the growth can be calculated as: 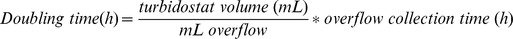



Measurement of room temperature fluorescence

Room temperature fluorescence was determined using a Dual PAM 100 (Walz, Effeltrich, Germany) with the emitter and detector units attached to the cuvette holder. Samples of 3 mL cells were stirred with a magnetic stirring bar at the bottom of a standard 1×1 cm glass cuvette. Each sample was transferred directly from the turbidostat to the cuvette and dark adapted for 15 min. whereupon F_V_/F_M_ was determined by a saturating pulse of 10000 µmol quanta m^−2^ s^−1^.

### Western blotting

Western blotting was performed as in [20]. Total protein extracts were separated in a 15% SDS-PAGE gel using CAH3 protein (Agrisera AB, Sweden) as an internal standard. Loading was done on an equal chlorophyll content (5 µg). Antibodies directed against *Chlamydomonas reinhardtii* CAH3 were obtained from AgriSera (product number AS05 073, Sweden) and used at a dilution of 1∶1000. After addition of secondary antibodies (BioRad, product number 172 1019) at a dilution of 1∶3000 and incubation for 90 s in freshly mixed peroxidase substrate (50% Immun-Star, Hrp-Peroxidase buffer (BioRad) and 50% Immun-Star HRP Luminol/Enhancer (BioRad)), the nitrocellulose membranes were covered with plastic wrap, exposed to X-ray film and developed.

### C_i_-response curves

C_i_-response curves were made by measurement of oxygen evolution in a Clark-type electrode (Hansatech). Cells were harvested and all inorganic carbon was washed out by centrifugation of the cells, discarding the supernatant and resuspending the pelleted cells in fresh C_i_-free BBM. This was repeated at least three times. The cells were then transferred to the oxygen electrode that was closed by the provided plunger and any residual C_i_ was removed by allowing photosynthesis to continue at an irradiance of 180 µmol quanta m^−2^ s^−1^ until net oxygen evolution was zero. At this time the light was switched off and respiration followed for a short time (2–3 minutes), the light was then switched on until no net photosynthesis occurred. This was considered to be the C_i_-free condition. Known amounts of NaHCO_3_ were added, starting at 0.5 µM and ending at 3.1 mM final concentration in the cuvette in 10 steps. In these experiments, the effects of inhibition of either only periplasmic CA or both periplasmic and chloroplastic CA activity was studied by addition of 50 µM acetazolamide (AZ) or 150 µM 6-ethoxy-2-benzo-thiazolesulfoamide (EZ), respectively, prior to the first addition of NaHCO_3_ [21]. The resulting C_i_-curves were modeled by non-linear regression (SigmaPlot 11, Systat Software, Inc.) using the Michaelis-Menten equation for determination of V_max_ and K_0.5_ (n = 4–6).

## Results

**Figure 1:**
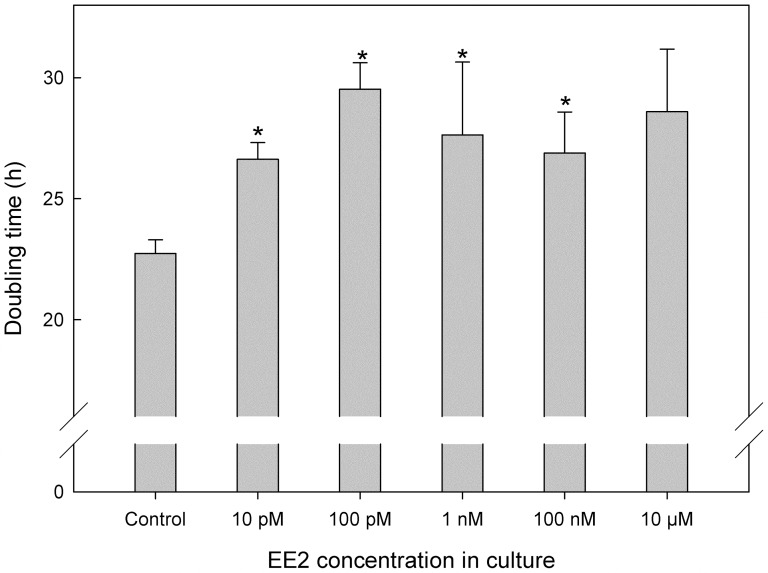
Growth doubling times for batch-grown cultures of *C. reinhardtii* grown in the presence of EE2 at concentrations ranging from 10 pM to 10 µM in the culture media. Control  =  no EE2 added. Growth was determined by daily measurements of A_750_, for a minimum of 10 days. Data represent means ± SE, n = 3. * above a bar indicates a significant change as compared to the control using a student's t-test and *p*<0.05.

Doubling times for the EE2-exposed cultures were determined for the batch and turbidostat grown cultures, respectively (Fig. 1, Table 1). As expected from previous experiments [22], the cells grown in batch cultures had higher doubling times than in the turbidostats. However, batch cultures were used to establish whether or not there was an effect on growth at all by EE2 as well as at which concentration the effect would become detectable. The turbidostat cultures were used to continuously provide cells growing at their maximum growth rate, μ_max_, for further experiments. It was shown that the growth rate was negatively affected at the lowest EE2 concentration tested, resulting in a change in doubling time from 22.7 h in the control to 26.6 h at 10 pM which is a significant increase by 17% (Fig. 1). Doubling times increased by further increasing the concentration of EE2 up to a concentration of 100 pM. At the highest concentration tested, 10 µM, the doubling time was 26% higher than in the control. Clearly, growth is negatively affected by EE2 additions.

**Table 1:** Growth doubling time for cultures of *C. reinhardtii* grown at high and low C_i_ without (control) and with addition of EE2 in the culture media.

Growth Condition	Doubling time (h)	
	Control	+ EE2
Low C_i_	15.8±2.3	a34.6±4.2
High C_i_	13.6±0.3	a22.8±4.2

Cultures were grown in turbidostats and the EE2 concentration was 7 µM. Data represent means ± SE, n = 4.

a indicates a significant change as compared to the control using a student's t-test and *p*<0.05.

Table 1 shows the doubling times for the turbidostat grown cultures with and without an active CCM as well as added EE2. The cultures with no addition of EE2 grew at the expected rates with the fastest doubling time, 13.6 h, for high C_i_-grown cells and 15.8 h for cells at low C_i_. Addition of EE2 increased the doubling time to 22.8 h at high C_i_ which was 68% higher than the control value, while at low C_i_ this was 34.6 h, which was 119% higher than the control level for low C_i_ grown cells (Table 2). Thus, similar to batch cultures, the addition of EE2 resulted in a pronounced effect on doubling times such that the growth of the cultures was severely slowed down and this effect was considerably stronger in low C_i_-grown cells where the CCM is expected to be fully active [23].

**Table 2:** Effects on F_V_/F_M_ on cells of *C. reinhardtii* grown at high and low C_i_ without (control) and with addition of EE2 in the culture media.

Growth Condition	F_V_/F_M_	
	Control	+ EE2
Low C_i_ grown	0.72±0.03	a0.65±0.03
High C_i_ grown	0.83±0.01	0.82±0.01

Cultures were grown in turbidostats and the EE2 concentration was 7 µM. Data represent means ± SE, n = 4.

a indicates a significant change as compared to the control using a student's t-test and *p*<0.05.

The parameter F_V_/F_M_ (Table 2) shows the potential maximum capacity for photochemistry and it is clear from the results that in cells grown at high C_i_, this is near or at the maximum [23] regardless if the cells were grown in the presence or absence of EE2. As expected, the control value for F_V_/F_M_ was 13.5% lower in low C_i_ than that for high C_i_ grown cultures (Table 2) and this reflects the activation of the CCM. Growth in the presence of EE2 and low C_i_ resulted in a significant and further 10% decrease in F_V_/F_M_ compared to the control cultures grown at low C_i_ (Table 2). This direct effect by EE2 on PS II photochemistry only in cells with an active CCM prompted a closer look at CA by Western blotting and photosynthesis parameters in the presence and absence of specific CA inhibitors.

**Figure 2:**
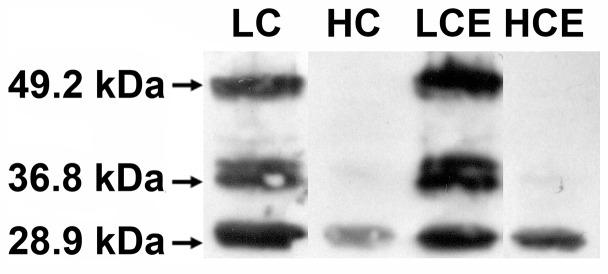
Immunoblot analysis of whole cell fractions from *C. reinhardtii* using a CAH3-specific antibody. Molecular mass markers are shown to the left. The four lanes (from left to right) are grown at: Low C_i_ (LC), High C_i_ (HC), Low C_i_ + EE2 (LCE) and High C_i_ + EE2 (HCE).

As expected, Western blots for the lumen-located carbonic anhydrase, CAH3, show that low C_i_ conditions resulted in a higher relative amount of CAH3 protein at 29.5 kDa compared to high-C_i_. However, we noted the presence of several additional bands (Fig. 2). The bands at approximately 38 and 49 kDa were detectable in both high, but especially in low-C_i_ grown cells (Fig. 2). The band at approximately 38 kDa appears to be a double band at slightly different size. It is unclear as yet if any of these additional bands are isozymes or dimers of the CAH3 but a 49 kDa band was present also in the internal standard (data not shown).

**Figure 3:**
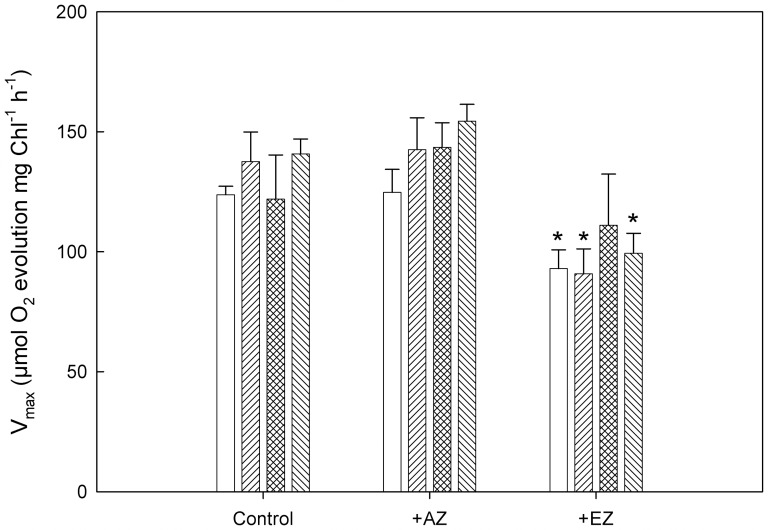
Change in maximal rate of photosynthetic oxygen evolution (V_max_) as determined from C_i_-response curves using the Michaelis-Menten equation for determination of V_max_. The bar legend is: no fill  =  Low C_i_ hatch fill diagonal left =  High C_i_, hatch fill both diagonals  =  Low C_i_ + EE2 and hatch fill diagonal right  =  High C_i_ + EE2. The irradiance during all measurements was 180 µmol quanta m^−2^ s^−1^ while the C_i_-response curves were determined by successive addition of HCO_3_^−^ from 0 up to 3.1 mM in 10 steps. Data represent means ± SE, n = 4–6. * above a bar indicates a significant change as compared to the control using a student's t-test and *p*<0.05.

**Figure 4:**
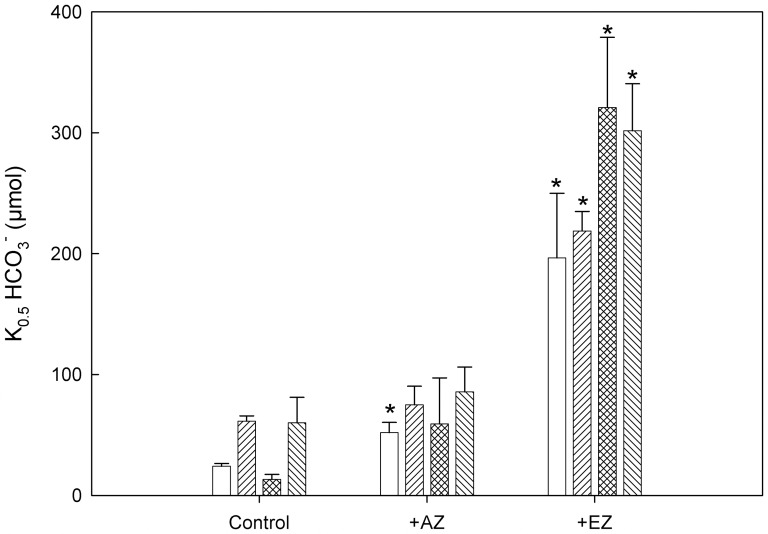
Change in the Michaelis constant, K_0.5_, determined as in **Fig. 3** from the Michaelis-Menten equation. Legend for bars as in Fig. 3. Error bars denote SE. n = 4–6. * above a bar indicates a significant change as compared to the control using a student's t-test and *p*<0.05.

Changes in photosynthetic V_max_ (Fig. 3) and changes in K_0.5_ (Fig. 4) for the cultures without and with added CA inhibitors were calculated from C_i_-response curves with data fitted to the Michaelis-Menten equation. There is no observable estrogen effect on V_max_ in the absence of CA inhibitors (control) or with the addition of AZ which inhibits CA activity in the periplasmic space (Fig. 3). This indicates that the maximum capacity for the assimilation of CO_2_ was unaffected by the inhibition of CAs of the CCM. However, in the presence of EZ, which inhibits CA activity both in the periplasmic space as well as in the chloroplast, a 30–35% reduction with little apparent changes between the different culture conditions was observed in V_max_ compared to the controls (Fig. 3).

As expected, a higher Michaelis-Menten constant, K_0.5_, was observed in the high-C_i_ grown cells (control) which indicates a lower affinity for CO_2_ both in the absence or presence of EE2 during growth (Fig. 4). A lower K_0.5_ was observed in cells from the low-C_i_ grown cultures which reflects an inducible higher affinity of the CAs for CO_2_ through activation of the CCM [15]. The C_i_ induced difference disappeared by the addition of AZ such that the K_0.5_ of low-C_i_ grown cells increased to the level of what the high-C_i_ grown cells displayed without the addition of AZ (Fig. 4). However, there is a pronounced and stronger effect on K_0.5_ for EE2-grown cells with EZ added (Fig. 4). The K_0.5_ increased to a level that was approximately 50% higher for the EE2-grown cells as compared to cells grown in the absence of EE2 regardless of if they were grown at low or high C_i_ (Fig. 4). This indicates that the chloroplastic CA activity is most sensitive to the effects of EE2.

## Discussion

To the best of our knowledge, this is the first time that an effect on algal growth has been shown to occur as a consequence of the synthetic estrogen hormone, EE2. The results clearly show an effect on the growth of *C. reinhardtii* under both high, but especially under low-C_i_-conditions when the estrogen EE2 was added to the cultures (Table 1). Furthermore, the effect was apparent at the low concentration of 10 pM, where a significant increase of doubling time by 17% was found, (Fig. 1) which is a level that is environmentally relevant [24], [25]. The doubling time increased further with further increase in EE2 concentrations. This effect was also manifested in a reduced maximal potential capacity of photochemistry as shown by a reduced F_V_/F_M_ in low C_i_ conditions with EE2 present during growth (Table 2). There is one recent report showing the effects on photosystem II by the addition of endocrine disrupting chemicals (EDC), however the EDC was added only 15 min. before the measurements were done [2]. Overall, the steroid estrogens appear to be the most potent EDCs of sewage effluent [4]. It has been known for some time that EDCs may be released by wastewater treatment plants in sufficient concentrations to cause vitellogenin biosynthesis in male fish, alterations of endocrine reproductive systems, decreased fertility and growth, poor hatching, egg shell thinning and abnormal thyroid function for birds, reptiles and mammals (reviewed in [26]). Removal of steroid estrogens in sewage treatment plants appears to vary in success from virtually complete removal to no removal at all and the poorest results are reported for colder, northern climates [4]. The suggested mechanism for the function of the EDCs released from sewage treatment plants is disruption of distribution of CA and CA activity in e.g. egg shell formation processes [12]. It is well established that *C. reinhardtii* expresses all three major CA evolutionary lineages [15]. To date, the only form of CA that appears to affect PS II is CAH3 and this inhibits photosynthesis and subsequent growth [14]. The reduced F_V_/F_M_ for cells grown under low C_i_ and with EE2 present during growth clearly indicates that EE2 directly affects the photochemistry in *C. reinhardtii* (Table 2). By using specific CA inhibitors on cells grown both in the presence and absence of EE2 under both high- and low-C_i_ conditions we could study the possible mechanism behind the effects of EE2 on algal growth. As compared to the control condition without inhibition of CA activity, there was only a limited effect on photosynthetic K_0.5_ by the addition of the external CA inhibitor AZ and no additional effect by EE2. (Fig. 4). The increase in K_0.5_ for the low-C_i_ grown cells in the presence of AZ can be attributed to the deactivation of the CCM [23]. However, the marked increase in K_0.5_ after addition of EZ to EE2 grown cells indicates that EE2 had affected chloroplastic CA by reducing the affinity for CO_2_ (Fig. 4). The main form of CA that affects photosynthetic efficiency, CAH3, is localized in the thylakoid membranes [27]. This enzyme has the ability to remove protons from the water splitting complex of photosystem II and by doing so will simultaneously provide HCO_3_^−^ to function as a proton carrier. Mutants lacking CAH3 are severely impaired in their photosynthetic capacity [14]. The increased relative abundance of CAH3 (29.5 kD) was expected [28] and observed under low C_i_ conditions regardless of whether the cultures were grown in the absence or presence of EE2 (Fig. 2). We also observed bands at approximately 38 and 49 kDa in low-C_i_ grown cells (Fig. 2). The band at 49 kD could indicate that CAH3 in *C. reinhardtii* is present as a dimer. At high-C_i_ these bands were suppressed so strongly that they were almost below detection, especially for the high-C_i_ grown cells with EE2 present. Future work is necessary to establish the significance of these additional bands. Interestingly, the blot suggests that there is more CAH3 present in both lanes with EE2 grown cells as compared to the lanes for the respective controls without EE2. The whole cell extracts of proteins used to produce the SDS-PAGE gels of separated proteins with subsequent blotting were loaded on a chlorophyll basis. Thus it is hard to, without further analysis, determine if the blot shows an actual difference. A possible explanation to the difference can be that EE2 triggers the cells to compensate for the inhibition by an increased production of CA.

Determination of V_max_ established that no specific effect could be seen in the EE2-treated cells (Fig. 3). This was not unexpected since the maximal rate was achieved at mM concentrations of HCO_3_^−^ added, thus reducing the importance of CA for C_i_ availability (see [29]).

## Conclusions

Our novel findings on the negative effects on green algal growth by the estrogen EE2 motivates further studies. In this study we have examined the effects on growth and photosynthesis in *C. reinhardtii* caused by the addition of EE2, but it would be important to also examine other estrogenic substances as well as other algae. Clearly, release of the estrogen EE2 in the waste water stream poses a potential problem not only with reduced removal of CO_2_ from the atmosphere but also a reduced growth rate which would have implications for the aquatic food web. Finally, these results indicate potential problems in using algae as a method to efficiently remove the inorganic nutrient load in waste water.

## Supporting Information

Figure S1 **The file is the unedited X-ray film of the Western blot used for** **figure 2** **.**
(JPG)

Dataset S1 **The file contains the datasets from the experimental work for this paper as an excel file with labeled tabs indicating which figure each set was used for.**
(XLS)
