# Supplementary figures and images for: Negative Impact on Growth and Photosynthesis in the Green Alga Chlamydomonas reinhardtii in the Presence of the Estrogen 17α-Ethynylestradiol
Source: PLoS One. 2014 Oct 13;9(10):e109289. doi: 10.1371/journal.pone.0109289 (PMC4195650; doi:10.1371/journal.pone.0109289)

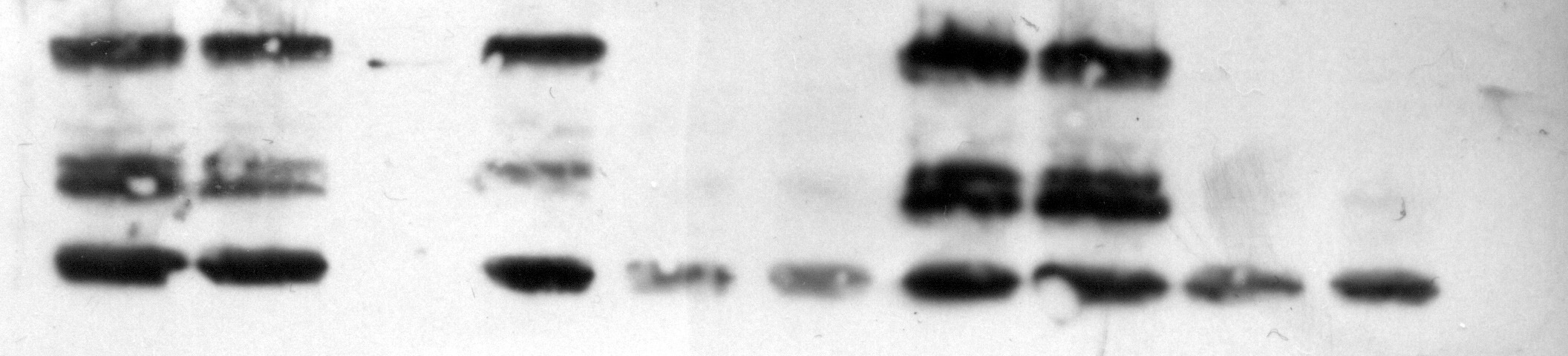

Supplement: Figure S1 — The file is the unedited X-ray film of the Western blot used for figure 2 . (JPG) [file pone.0109289.s001.jpg]
